# A case of adult-onset Wolfram syndrome with compound heterozygous mutations of the WFS1 gene

**DOI:** 10.1016/j.ajoc.2022.101315

**Published:** 2022-01-22

**Authors:** Jinhee Lee, Takuya Iwasaki, Tomoko Kaida, Hideki Chuman, Akiko Yoshimura, Yuji Okamoto, Hiroshi Takashima, Kazunori Miyata

**Affiliations:** a Miyata Eye Hospital, Miyakonojo, Miyazaki, Japan; b Department of Ophthalmology, Faculty of Medicine, University of Miyazaki, Miyazaki, Japan; c Department of Neurology and Geriatrics, Graduate School of Medicine and Dental Sciences, Kagoshima University, Japan; d Department of Physical Therapy, School of Health Sciences, Faculty of Medicine, Kagoshima University, Kagoshima, Japan

**Keywords:** Wolfram syndrome, Adult-onset, Compound heterozygous mutation

## Abstract

**Purpose:**

Wolfram syndrome is a rare genetic disorder characterized by juvenile onset of diabetes mellitus with bilateral optic atrophy. We report a case of adult onset Wolfram syndrome with diabetes mellitus at age 22 and optic atrophy after age 40. The WFS1 gene sequence was analyzed in the patient and her father.

**Observations:**

A 46-year-old woman presented with bilateral vision loss. She had developed diabetes mellitus at age 22 and underwent bilateral cataract surgery at age 37. Visual acuity was 20/50 in the right eye and 20/200 in the left eye. The pupillary light reflex was sluggish in both eyes. Fundus examination showed bilateral optic atrophy, but there was no diabetic retinopathy. Cecocentral scotoma of both eyes was observed in Goldmann perimetry. There were no intracranial lesions on magnetic resonance imaging. Audiometry demonstrated high-frequency sensorineural hearing loss. Sequence analysis of the WFS1 gene revealed compound heterozygous mutation: c.908T>C p.L303P and c.1232_1233del, p.S411Cfs*131 in the patient and heterozygous mutation c. 908 T>C, p. L303P in her father.

**Conclusions and importance:**

The patient was diagnosed with adult-onset Wolfram syndrome with compound heterozygous mutations of the WFS1 alleles. Wolfram syndrome must be ruled out even in adult-onset diabetic patients with optic atrophy.

## 1 Introduction

Wolfram syndrome is an autosomal recessive disorder characterized by juvenile onset diabetes mellitus and bilateral optic atrophy, followed by diabetes insipidus, sensorineural deafness, urinary tract abnormalities, and ataxia and other neurodegenerative disorders.^1^ The main pathology of Wolfram syndrome is loss of the insulin-secreting cells in the pancreas, retinal ganglion cells, and myelinated axons in the optic nerves.^2^^,^^3^ The incidence of Wolfram syndrome is estimated to be one in 770,000 in the United Kingdom^4^ and one in 710,000 in Japan.^5^ Wolfram syndrome has been demonstrated to result from mutations in two loci: WFS1 and WFS2/CISD2, localized on chromosome 4p16^6^^,^^7^ and 4q24,^8^ respectively.

WFS1 mutations are also reportedly linked with various autosomal dominant diseases of low-frequency hearing loss,^9^ optic atrophy,^10^^,^^11^ and insulin-dependent diabetes mellitus.^12^ The WFS1 gene-encoded protein wolframin is localized in the endoplasmic reticulum and consists of 3 domains: an amino-terminal hydrophilic cytoplasmic domain, a hydrophobic region containing 9 transmembrane segments, and a carboxy-terminal hydrophilic endoplasmic domain. It is highly expressed in the nervous tissue and pancreas.^13^

Though typical cases of Wolfram syndrome develop the clinical symptoms in childhood, we report a patient with a compound heterozygous mutation of the WFS1 gene and adult-onset diabetes mellitus and optic atrophy.

## 2 Case report

**Fig. 1:**
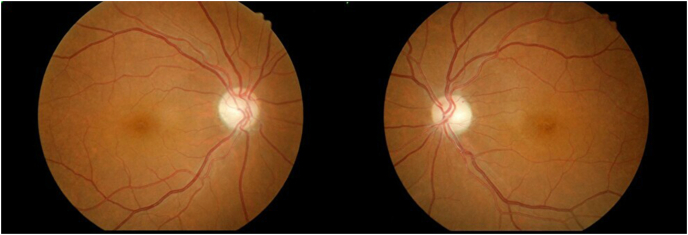
The fundus photographs. Fundus photographs show a normal retina and optic atrophy in both eyes.

**Fig. 2:**
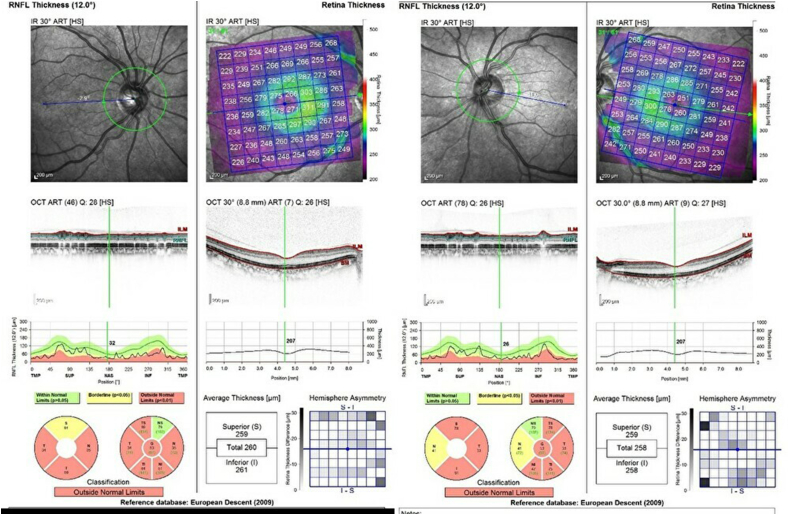
The optical coherence tomography. The optical coherence tomography shows decreased retinal fiber layer thickness in both eyes.

**Fig. 3:**
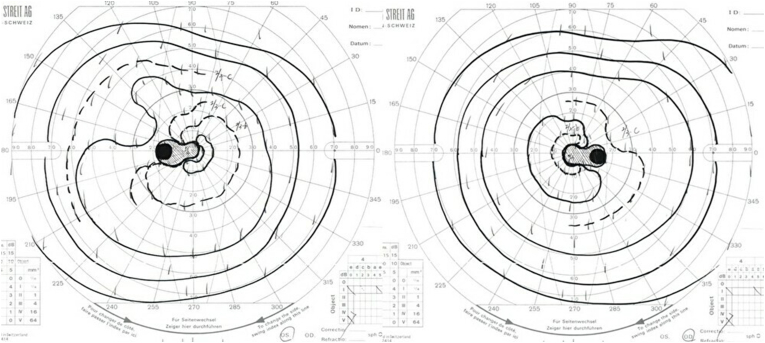
The visual fields. Goldmann perimetry demonstrates cecocentral scotoma in both eyes.

**Fig. 4:**
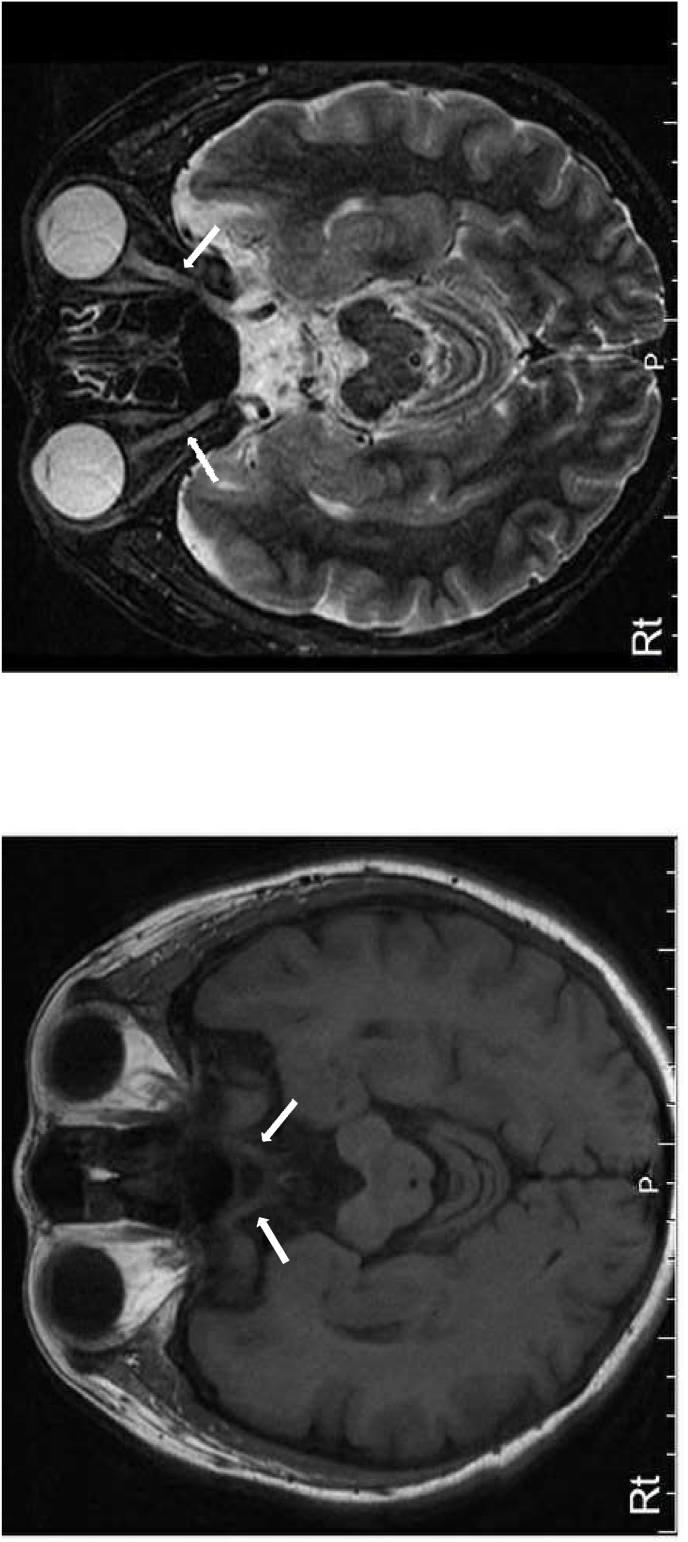
The magnetic resonance imaging of the optic tract and optic chiasm. Magnetic resonance imaging demonstrates bilateral optic atrophy from the optic nerves (arrows) to the optic tract. Right panel: Fluid-attenuated inversion recovery (FLAIR); Left panel: short TI inversion recovery (STIR). Rt: Right.

A 46-year-old woman presented with vision loss in both eyes. She was diagnosed with diabetes mellitus at age 22 and had undergone bilateral cataract surgery at age 37. She was treated with insulin therapy, but she frequently was noncompliant insulin injections. Her hemoglobin A1c was 10.5, she was negative for anti-GAD antibodies, and had good renal function. She had been smoking around half a pack per day for three years and had no history of alcohol or recreational drug use. There was no family history of similar illness. Her best-corrected visual acuity was 20/50 in the right eye and 20/200 in the left eye. Her pupil sizes were equal and light reflex was sluggish. The ocular position and external ocular movement were normal. No abnormality was found in the anterior segment and optic media. Normal retinas and optic atrophy were found in fundus examination (Fig. 1). Optical coherence tomography revealed decreased retinal fiber layer thickness (Fig. 2). Goldmann perimetry demonstrated cecocentral scotoma in both eyes (Fig. 3). Magnetic resonance imaging demonstrated optic atrophy from the optic chiasm to the optic tract but did not show any causative lesion (Fig. 4). A hearing test showed symmetric high-frequency sensorineural hearing loss.

**Fig. 5:**
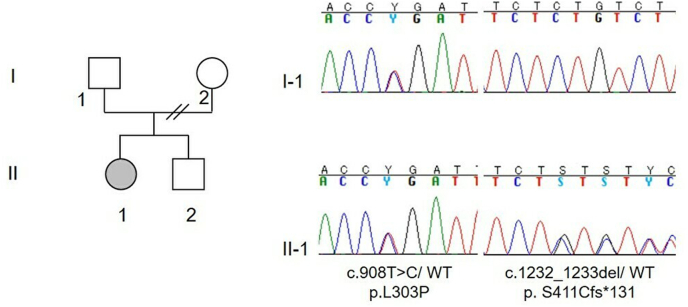
WFS1 gene sequence analysis of the patient (II-1) and her father (I-1). A heterozygous c.908T>C mutation is found both in the patient and her father, and a heterozygous c.1232 = 1233 deletion was only observed in the patient. WT: wild type.

After written informed consent was obtained, genetic analysis was performed for the patient and her father. The protocol for genomic analysis was reviewed and approved by the institutional review board. Initially, we performed whole exome sequencing followed by a directed confirmation of the identified WFS1 pathogenic variants by sanger sequencing. Compound heterozygous mutations were found in exon 8 of the patient's WFS1 gene; one allele was a missense mutation: c. 908 T>C, p. L303P, and the other allele was a frameshift mutation: c.1232_1233del, p.S411Cfs*131. Her father had heterozygous mutation c. 908 T>C, p. L303P. Genetic analysis could not be performed for her mother (Fig. 5). The patient's compound heterozygous WFS1 mutations were confirmed by specially designed allele-specific primer sets. Using the official criteria for Wolfram syndrome, which include early onset diabetes under the age of 30, optic atrophy, and detection of molecular mutations in the WFS1 gene,^14^ she was diagnosed with Wolfram syndrome.

## 3 Discussion

This case of Wolfram syndrome was unusual as the patient developed diabetes mellitus after age 20 and the clinical manifestations of optic atrophy only became apparent after age 40. Diabetes mellitus develops by the age of 20 in 94% of patients with Wolfram syndrome, and visual impairment due to optic atrophy is seen in 98% of patients under 40 years old.^15^

Because various mutations have been detected in the WFS1 gene, the correlation between the WFS1 genotype and the Wolfram syndrome phenotype has not been clearly defined.^15^ WFS1 gene mutations were detected in approximately 68% of Japanese patients with Wolfram syndrome (n = 30), including 70% with homozygous mutations and 30% with compound heterozygous mutations.^5^ Genetic analysis of the WFS1 allele in the present case revealed a compound heterozygous mutation, c. 908 T>C, p. L303P and c.1232_1233del, p.S411Cfs*131. The p.S411Cfs*131 mutation, in the transmembrane domains of wolframin, has been reported in Wolfram syndrome.^16^ However, the c.908T>C p.L303P mutation, in the cytoplasmic domain, has been reported in one kindred with compound heterozygous mutations with c.1245ins (TCT) and in one allele of two kindred children with hearing loss and without diabetes mellitus and optic atrophy.^17^ The compound heterozygous mutation of the WS1 gene in the present case might delay the onset of clinical manifestations. However, we could not find any reports in the literature addressing the effect of compound heterozygous mutations on the onset of clinical manifestations.

In the present case, the age of onset of diabetes mellitus was 22 years and anti-GAD antibody was not detected. Although the patient was treated with insulin, she frequently skipped insulin injections and never develop hyperglycemic coma. Diabetes mellitus in typical cases of Wolfram syndrome is insulin-dependent without anti-GAD antibody.^4^ The apparent lack of insulin dependence in the present case might be related to the number of remnant insulin-secreting cells in the pancreas. The present case seemed to progress more slowly than the typical clinical course of Wolfram disease.

## 4 Conclusions

Wolfram syndrome should be considered if optic atrophy is confirmed in patients with diabetes mellitus. This case illustrates the importance of WFS1 gene sequencing in diabetic patients with bilateral optic atrophy, even in those greater than 20 years of age at onset. The increased availability of genetic testing will enable identification of more genetic mutations, help to clarify the relationship between the homozygous and heterozygous mutations of WFS1, and aid in finding interventions to delay the onset of various pathology in Wolfram syndrome patients.

## Patient consent

We have obtained the patient's written consent for the publication. This report does not contain any personal information that could lead to the identification of the patient.

## Funding

Applying Health and Technology (201331010B) of Ministry of Health, Welfare and Labour, Japan.

No funding was received for this work.

## Intellectual property

We confirm that we have given due consideration to the protection of intellectual property associated with this work and that there are no impediments to publication, including the timing of publication, with respect to intellectual property. In so doing we confirm that we have followed the regulations of our institutions concerning intellectual property.

## Research ethics

We further confirm that any aspect of the work covered in this manuscript that has involved human patients has been conducted with the ethical approval of all relevant bodies and that such approvals are acknowledged within the manuscript.

IRB approval was obtained (required for studies and series of 3 or more cases).

Written consent to publish potentially identifying information, such as details or the case and photographs, was obtained from the patient(s) or their legal guardian(s).

## Authorship

The International Committee of Medical Journal Editors (ICMJE) recommends that authorship be based on the following four criteria:

1. Substantial contributions to the conception or design of the work; or the acquisition, analysis, or interpretation of data for the work; AND
2. Drafting the work or revising it critically for important intellectual content; AND
3. Final approval of the version to be published; AND
4. Agreement to be accountable for all aspects of the work in ensuring that questions related to the accuracy or integrity of any part of the work are appropriately investigated and resolved.

All those designated as authors should meet all four criteria for authorship, and all who meet the four criteria should be identified as authors. For more information on authorship, please see http://www.icmje.org/recommendations/browse/roles-and-responsibilities/defining-the-role-of-authors-and-contributors.html#two.

All listed authors meet the ICMJE criteria.  We attest that all authors contributed significantly to the creation of this manuscript, each having fulfilled criteria as established by the ICMJE.

One or more listed authors do(es) not meet the ICMJE criteria.

We believe these individuals should be listed as authors because:

We confirm that the manuscript has been read and approved by all named authors.

We confirm that the order of authors listed in the manuscript has been approved by all named authors.

## Declaration of competing interest

No conflict of interest exists.
